# Comprehensive profiling of rRNA-derived small RNAs in *Arabidopsis thaliana* using rsRNAfinder pipeline

**DOI:** 10.1016/j.mex.2023.102494

**Published:** 2023-11-25

**Authors:** Garima Kalakoti, AT Vivek, Anshul Kamboj, Ajeet Singh, Srija Chakraborty, Shailesh Kumar

**Affiliations:** a Bioinformatics Laboratory, National Institute of Plant Genome Research (NIPGR), Aruna Asaf Ali Marg, New Delhi 110067, India; b Department of Ophthalmology, Baylor College of Medicine, Houston, TX, United States

**Keywords:** Ribosomal RNA, non-coding RNA, rRNA derived small RNAs, sRNA-seq, *Arabidopsis thaliana*, rsRNAfinder

## Abstract

Ribosomal RNA (rRNA) gives rise to non-random small RNA fragments known as ribosomal-derived small RNAs (rsRNAs), which despite their biological importance, have been relatively understudied in comparison to other short non-coding RNAs. There exists a compelling necessity to develop a methodology for the identification, categorization, and quantification of rsRNAs from small RNA sequencing (sRNA-seq) data sets, considering the unique characteristics of ribosomal RNA (rRNA). To bridge this gap, we introduce ‘rsRNAfinder’ a specialized pipeline designed within the Snakemake framework. This analytical approach enables robust identification of rsRNAs using sRNA-seq datasets from *Arabidopsis thaliana*. Our methodology constitutes an integrated bioinformatic pipeline designed for different kinds of analysis.

1. **sRNA-seq data analysis**: It performs in-depth analysis of reference-aligned sRNA-seq data, facilitating rsRNA annotation and quantification.
2. **Parametric reporting**: Our pipeline provides comprehensive reports encompassing key parameters such as rsRNA size distributions, strandedness, genomic origin, and source rRNA origin.
3. **Illustrative validation**: We have demonstrated the utility of our approach by conducting comprehensive rsRNA annotation in *Arabidopsis thaliana*. This validation reveals unique rsRNAs originating from all rRNA types, each of them distinguished by distinct identity, abundance, and length.

## Graphical abstract

**Figure:**
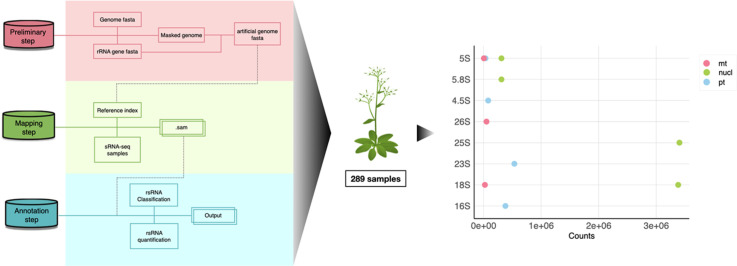

Specifications table

**Table:** 

Subject area:	Bioinformatics
More specific subject area:	RNAinformatics
Name of your method:	rsRNAfinder
Name and reference of original method:	N.A.
Resource availability:	https://github.com/rebminso/rsRNAfinder

## Background

In recent years, the field of non-coding RNA research has made significant progress, shedding light on the potential roles of small RNAs in gene regulation, transcription, and translation [1], [2], [3], [4]. The emergence of Next Generation Sequencing (NGS) technologies has greatly contributed to the identification of diverse classes of long and short non-coding RNAs [5]. Among the various types of cellular RNAs, rRNA stands out as the most abundant, surpassing both mRNA and tRNA. Similar to tRNAs, rRNA can undergo fragmentation, leading to the formation of smaller fragments known as rsRNAs. These rsRNAs have been detected in various organisms, and accumulating evidence suggests that their cleavage is not random but rather occurs with precision [6], [7], [8], [9], [10]. However, compared to tRNA-derived small RNAs (tsRNAs), our understanding of rsRNAs remains limited. Given the rise of NGS technologies and growing interest in the role of non-coding RNA in gene regulation, the investigation of rsRNAs has become increasingly relevant [10], [11], [12], [13]. Therefore, it is crucial to develop a method for the identification and classification of this understudied class of ncRNA to gain a deeper understanding of the molecular mechanisms underlying cellular processes.

In this study, we developed a new method using the Snakemake workflow management system [14], to identify high-confidence rsRNAs. By analyzing published sRNA-seq data from *Arabidopsis thaliana*, we investigated the distribution and properties of rsRNAs in different tissues. Our analysis revealed unique characteristics, abundance levels, and tissue-specific patterns of rsRNAs. We also examined the modification sites within rsRNAs and explored their potential roles in known pathways. Furthermore, we compared rsRNA profiles between mutant and wild-type samples, highlighting the involvement of DCL and FRY genes in rsRNA biogenesis. These findings serve as a valuable resource for further studies on the biological functions of rsRNAs, with implications for research on other species.

**Method details**

## Overview of rsRNA identification pipeline

The implementation of rsRNAfinder is structured as a set of rules within a workflow, connecting input files to output files. Each rule represents a specific action, and upon execution, the workflow wisely selects the most efficient combination of rules to generate the desired output. This optimization ensures that the pipeline runs as efficiently as possible to complete the assigned tasks. Users can interact with rsRNAfinder through a single configuration file, where they can specify the paths to their input FASTQ files, reference genome, and relevant analysis parameters in a human readable YAML format. Running the entire pipeline is simplified to a single command, and it generates two main folders: ‘an intermediate directory’ and ‘a result directory’. These directories contain well-organized subfolders that host the intermediate files of analysis, making it easy for users to navigate and access their results. One notable advantage of rsRNAfinder is its ability to efficiently re-run the analyses. If the input files remain unchanged, the pipeline avoids re-executing upstream steps, which saves time and computational resources. This feature is especially useful for error correction, data sub-setting, or parameter adjustments.

The overall rsRNAfinder pipeline comprised of the construction of an artificial genome; alignment of small RNA-seq reads against the pre-build reference, identification, classification, and visualisation of rsRNAs, providing insights into their expression levels and patterns. The filtered small RNA reads, mapped to the artificial genome, facilitating the determination of their origin and precise location. Subsequently, rsRNAs are identified, classified, and categorized based on their unique characteristics and functions. Lastly, rsRNAfinder quantifies the abundance of rsRNAs, providing insights into their expression levels and patterns. Each step of the workflow is described in detail below.

### Construction of artificial genome

To enable the detection of rsRNAs from sequencing reads, an artificial genome was created by masking the rRNA genes, with or without flanking regions in the reference genome. This artificial genome, along with the rRNA sequences, was subjected to processing using ‘segemehl’ aligner (v0.12.8) [15]. During this step, maximum of two differences in the seed were allowed and the maximum number of reads mapping to the same genomic position was set to 50. The resulting indexed files and artificial genome facilitated the identification of rsRNAs from the sequencing data. This step determined the origin and precise location of the reads, providing crucial information for downstream analysis. It is important to note that the default sequence used in this procedure was obtained from NCBI.

### Identification and classification

Only reads that exclusively mapped to the rRNA regions were retained for subsequent analysis, to prevent ambiguous reads from non-rRNA regions. This step helps to categorize rsRNAs into distinct groups. Based on their specific mapping positions, the selected reads were categorized as rRNA-derived fragments (rRFs) namely rRF-3, rRF-5, or rRF-i. Stringency criteria prioritized rRFs with a defined number of mismatches or indels in the alignment.

### Quantification and estimation

To standardize the comparison of rsRNA expression levels in a sample, the abundance of each rsRNA is normalized to reads per million (RPM). Stringent parameters were applied during the analysis to ensure accurate quantification and estimation. Specifically, confident rsRNAs were identified using a threshold of less than 4 differences (indels and mismatches) and a minimum abundance of 10 RPM. In cases where multiple rsRNAs were located at the same genomic positions but exhibited low abundance, they were excluded, while still maintaining the overall count of rsRNAs. This filtering step aimed to prioritize rsRNAs with higher abundance, ensuring a precise representation of their collective expression levels.

### Output

**Fig. 1:**
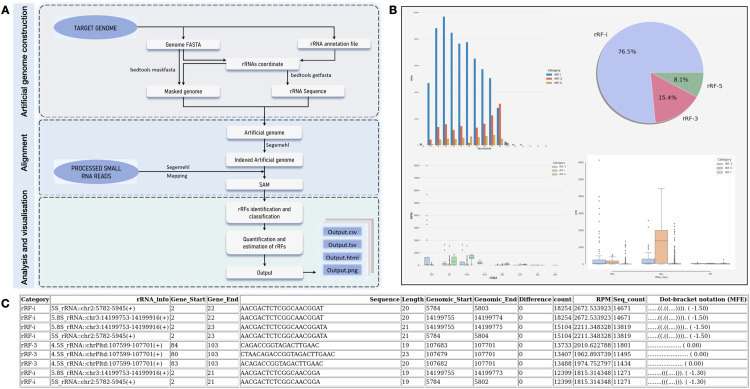
Overview of rsRNAfinder workflow using sRNA-seq data**.** A) Schematic outline of rsRNAfinder workflow, encompassing artificial genome creation, small RNA sequencing data alignment using segemehl algorithm, subsequent analysis, and results visualization. B) Integrated libraries within rsRNAfinder produce informative figures, including pie charts, box plots, and bar plots, depicting distribution and characteristics of rsRNAs. C) rsRNAfinder generates an HTML-formatted analysis report for user-friendly data exploration and interpretation.

The workflow generates tab-delimited and HTML output files that provide analysis results for rsRNAs. These results include information on rsRNA length, sequence, counts, and positions. Additionally, the output includes category-wise counts of rsRNA, abundance statistics, and plots that depict the distribution of rsRNAs based on their organellar origin. The toolkit integrates libraries such as ‘pandas’, ‘matplotlib’, and ‘Seaborn’ to facilitate visualization of the output generated from each sample. Each step of the pipeline is implemented as a customizable function within ‘Snakemake’, enabling efficient customization and adaptation. Fig. 1A provides a conceptual overview of the pipeline workflow. We categorized rsRNAs, which range from 16 to 40 nucleotides in length, into three primary types based on their reference to cognate rRNA sequences, as previously applied classification system [7]. These categories include 5′-rRFs, which originate from the first position of the rRNA transcript; 3′-rRFs, which terminate at the final position of the rRNA transcript, representing the 3′-end regions; and i-rRFs, characterized by their internal start and end positions within the rRNA transcript. We followed this approach since studies have shown that rsRNAs are produced in a regimented manner by unknown processes and it helps in understanding the pattern with which rsRNAs are generated from parental rRNAs [10,7].

## rsRNAfinder pipeline usage

For users interested in exploring and utilizing the rsRNAfinder tool for robust rsRNA analysis, the complete source code and a detailed overview are available on GitHub (https://github.com/rebminso/rsRNAfinder.git), and it is designed to be compatible with various operating systems, including Windows, Linux, and MacOS.

To start using the rsRNAfinder pipeline for analyzing sRNA-seq datasets, follow these steps:

A. Repository Download and Dependencies:

Clone github repository (https://github.com/rebminso/rsRNAfinder.git). Setup conda environment. Snakemake manages additional dependencies automatically, provided it is installed properly. The repository has a primary directory named "rsRNAfinder" with several subfolders.

B. Configure Workflow:

Customize the configuration of workflow by editing the “config/config.yaml” file. There can be two scenarios as indicated below-

a. Using *Arabidopsis thaliana* as the reference genome (no changes needed in the config file).
  i. Add trimmed FASTQ file folder to the data/trimmed/ directory (follow filename format).
b. Using a different genome:
  i. Add trimmed FASTQ file folder to the data/trimmed/ directory (follow filename format).
  ii. Include the FASTA sequence of the reference genome in the data/Genome/ directory.
  iii. Add the genome feature table in .txt format to the data/Feature_table/ directory (NCBI source only).
c. Modify the Config file to specify the reference genome, genome fasta file path, and species NCBI feature table path as follows:
  i. Search strategy: "host"
  ii. genome_fasta: path/to/genome/file
  iii. species_NCBI_feature_table: path/to/NCBI/feature/table

Edit the genome file to format the headers correctly. Each genome fasta header should start with >chr[N*um*]'. Mitochondrial and plastid fasta headers should be as 'chrMt' and 'chrPt,' respectively.

C. Run the Workflow:

The workflow can be executed after proper configuration and deployment, with the current working directory set to “∼./rsRNAfinder”. To run, the following command should be executed:

snakemake –cores 8 -q

-q can be replaced with -npr to perform a dry run of the workflow. The main Snakefile in the “./rsRNAfinder” directory will be automatically detected, and all steps will be executed. You can also increase the number of cores as needed.

D. Results:

The “intermediate/” and “result/” directories are created in the current working directory, specifically in “./rsRNAfinder.” The “intermediate/” directory houses files generated during processing, while the “result/” directory contains the output files. Each input sample is allocated a separate folder within a directory named after the input reference genome. Upon completion of the process, the “result” directory will encompass various files, including:

a. A comma separated file (CSV) with information on the identified rsRNAs, including details about the category of respective rRNAs, such as length, sequence, raw and normalized counts, gene start and end, and genomic start and end positions of rRFs. This file contains columns of information as represented in Table 1:

**Table 1:** Overview of column information for annotated rsRNAs.

Columns	Description
Category	class of rRFs based on the mapping position of the read on the genome.
rRNA_info	general rRNA information such as parental coordinates and chromosome number.
Gene_Start	coordinate where the first base of the read maps to with respect to the gene.
Gene End	coordinate where the last base of the read maps to with respect to the gene.
Sequence	mapped nucleotide sequence.
Length	length of the mapped bases
Genomic Start	coordinate where the first base of the read maps to with respect to the genomic position.
Genomic End	coordinate where the first base of the read maps to with respect to the genomic position.
Difference	sum of total number of mismatches, insertions, and differences in the mapped sequence.
RPM	ratio of the reads supporting the rRFs to the total number of small RNA sequences.
Seq_count	count of each unique sequence mapping to each genomic locus.
count	count of reads mapping exactly to the same genomic locus with some differences allowed.
b. An eight-column file for abundance statistics summarizes the most abundant rsRNAs for each rRNA type. The toolkit has several integrated libraries for quick visualization of the output from each sample. These plots provide a better representation of the distribution of the results, including a pie plot, a bar plot, and two box plots (Fig. 1B).
c. A .tsv file with two columns showing the count of occurrence of each rsRNA class in the sample.
d. html: This presents the data on a webpage. The abundant rRFs from individual genomic locations are considered and the count of all mapped reads is kept in the creation of the HTML file from the above CSV file with an additional column of dot-bracket notation. The reads supporting the rRFs are divided by the total number of single sRNA seq and then multiplying with 1 million to normalize the rRF abundance to RPM (Fig. 1C).
E. Test run:

**Download and configure rsRNAfinder**

Clone the rsRNAfinder repository from GitHub and navigate to the rsRNAfinder directory:

git clonehttps://github.com/rebminso/rsRNAfinder.git
unzip rsRNAfinder
cd rsRNAfinder

Activate the base conda environment:

conda activate base

Create and activate the rsRNAfinder conda environment using the provided environment.yml file:

conda env create -f config/environment.yml
conda activate rsRNA

**An example using a default genome i.e. *Arabidopsis thaliana***

### Download the input and genome file

• A test folder containing an input file is provided in ‘data/trimmed/’ directory.

gunzip data/trimmed/test/*

• Genome file is already provided in ‘data/Genome/’ directory.

gunzip data/Genome/*

### Run the test sample

snakemake –cores 8 -q

-q can be replaced with -npr to perform a dry-run. These steps will enable you to perform a test run of rsRNAfinder using the default genome (Arabidopsis thaliana) and the provided test input data.

**An example using any other genome**

### Download input sample

Download single-end scRNA-seq input reads from the SRA database. Store the downloaded sequences in the “path/data/trimmed/” folder.

cd path/data/
mkdir newfq
cd newfq
fastq-dump SRR24973537

### Download the genome file of interest

Download the genome file for your target organism. Example: *Homo sapiens* (GRCh38.p14).

cd pathof/data/Genome
wgethttps://ftp.ncbi.nlm.nih.gov/genomes/refseq/vertebrate_mammalian/Homo_sapiens/latest_assembly_versions/GCF_000001405.40_GRCh38.p14/GCF_000001405.40_GRCh38.p14_genomic.fna.gz
gunzip *
mv GCF_000001405.40_GRCh38.p14_genomic.fna hs.fna

### Download feature table file of interest

Download the feature table file for your target organism. Example: *Homo sapiens* (GRCh38.p14).

cd pathof/data/
mkdir Feature_table
cd Feature_table
wgethttps://ftp.ncbi.nlm.nih.gov/genomes/refseq/vertebrate_mammalian/Homo_sapiens/latest_assembly_versions/GCF_000001405.40_GRCh38.p14/GCF_000001405.40_GRCh38.p14_feature_table.txt.gz
gunzip *
mv GCF_000001405.40_GRCh38.p14_feature_table.txt hs.txt

### Update the config file

reference_genome:
Search_strategy: "host"
genome_fasta: "data/Genome/hs.fna"
species_NCBI_feature_table: "data/Feature_table/hs.txt"

### Run rsRNAfinder

Return to the rsRNAfinder directory and execute the following command to run the test sample:

snakemake –cores 8 -q

-q can be replaced with -npr to perform a dry-run of the workflow. Make sure to replace “pathof/data/” with the actual path to your data directory in the instructions.

## Method validation

The approach was applied to a large set of sRNA-seq datasets described below.

**(A) *Arabidopsis thaliana*Col-0 ecotype libraries:** We applied rsRNAfinder to analyze sRNA-seq data obtained from various tissues of *Arabidopsis thaliana*, specifically the Col-0 ecotype, with the aim of understanding their expression patterns. Tissue samples were assigned to distinct groups based on their origin or shared morphology, including flower, fruit, leaf, shoot, seed, and root. We selected specific datasets with adequate raw data availability to ensure robust sequencing depth (>1 million mapped reads), and uniform conditions for adapter trimming and read mapping. Specific details regarding the origin and accession of the sRNA-seq data can be found in Table S1. To trim the reads across all small RNA-seq datasets, TrimGalore (v0.0.4) (https://github.com/FelixKrueger/TrimGalore) was utilized, applying a minimum quality threshold of 20. The resulting trimmed reads were aligned to the *A. thaliana* genome (version TAIR10) using Segemehl with the options -D 2 and -M 50. For the alignment procedure, we allowed 1 to 3 nucleotide mismatches over the mapped length, with a maximum mismatch rate of 5 %. The final identification of rsRNAs was based on determining the predominant read sequence at each locus within the parent rRNA.

All the steps above are part of rsRNAfinder tool except the trimming step of raw reads. In addition, we conducted an analysis into snoRNA-based modifications by searching the snOPY [16] for modification site data on target rRNAs to identify possible rsRNAs that have retained the modifications. To predict rsRNA target genes, we employed psRNATarget [17] with Araport11 [18] annotated transcript sequences. This was done with the assumption that rsRNA might also have a similar regulatory role similar to that of miRNAs and tsRNAs. For gene target prediction, we utilized Schema V2, considering only the top two hits for each target. To assess the potential functions of the predicted rsRNA-targeting genes, we performed pathway enrichment analysis. Additionally, for confirmation of rsRNA-targets, Cleaveland (v4.5) [19] was employed with default parameters, utilizing data from three Parallel Analysis of RNA Ends sequencing (PARE-Seq) datasets: GSM1263708 (flower), GSM1330569 (leaf), and GSM3321205 (seedling).

**(B) mutant libraries:** To determine whether the biogenesis of rsRNA loci could be attributed to established sRNA biogenesis mechanisms [[3], [4], [5],^19^], we examined rsRNA expression in mutants related to sRNA biogenesis using publicly available sRNA-seq libraries from *dcl, rrp6l*, and *fry* mutant plants (details in Table S2). Following the previously described steps in (A), both the mutant and wild-type libraries were subjected to trimming and alignment against the *A. thaliana* genome using rsRNAfinder. The resulting output files from these analyses were subsequently used for further interpretation.

### Discovery and annotation of rsRNAs in multiple tissues of *Arabidopsis thaliana*

In the sRNA-seq data utilized for this study, we observed variation in the average alignment rate to the *Arabidopsis* genome among the samples, with an average rate of 86.52 %, as depicted in Figure S1A. Notably, approximately 30 % of the total sRNA-seq reads mapped to the reference rRNA. Furthermore, the mapping percentages exhibited variations corresponding to distinct tissue types, as illustrated in Figure S1B. These findings collectively laid a foundation for the comprehensive identification of rsRNAs and facilitated the comparison of their normalized abundances across all sRNA-seq libraries.

By utilizing this adopted workflow, we were able to successfully annotate a considerable number of distinct rsRNAs, suggesting a modulation of rsRNA production that appears to be influenced by the specific tissue type and state. We identified a substantial number of unique rsRNAs based on their position within rRNA transcripts and unique sequences. This exhaustive cataloging of rsRNAs was achieved despite the challenges posed by extensive multi-mapping to the reference genome. Figure S2 depicts the counts of unique rsRNAs exceeding the threshold of >10 RPM in the analyzed datasets, demonstrating a consistent processing rate across rRNA transcripts. Further, the mapping data to the artificial genome strongly suggests that most of them are exclusive (∼73 %) to rRNA space. Thus, the use of an artificial genome enabled us to estimate the degree of ambiguity based on those mapping to non-rRNA space as well. Additional analysis showed a widespread distribution of rsRNAs across samples, with certain rsRNAs detected in more than multiple samples, while others exhibited limited occurrence in a subset of samples. Notably, no rsRNA was identified to be consistently present in all samples, indicating a diverse and context-dependent landscape of rsRNA populations. Despite these results emphasizing the heterogeneity and complexity of rsRNA profiles, it is crucial to be cautious of the presence of noise in the expression results. Such noise could be tackled by corroborating expression levels in samples of similar biotype if needed and/or by using other normalization methods.

The distribution of rsRNAs was predominantly skewed towards 5′ rRFs, and we also observed a significant abundance of rsRNAs derived from all rRNAs in the length range of 16–30 nucleotides (Figure S2). However, there were no major differences in the overall relative size distribution of rsRNAs derived from nuclear, mitochondrial, or chloroplastic-encoded rRNA, respectively. On the other hand, there were significant differences in the sizes of fragments originating from nuclear, mitochondrial, and chloroplast-encoded rsRNAs, respectively. We found that less than one-quarter of rsRNAs in *Arabidopsis* were derived from organellar rRNAs, as evident in Fig. 3a. However, it remains uncertain whether rsRNAs are biologically generated from mitochondria, as they exhibited exceptionally lower abundance compared to rsRNAs.

**Fig. 2:**
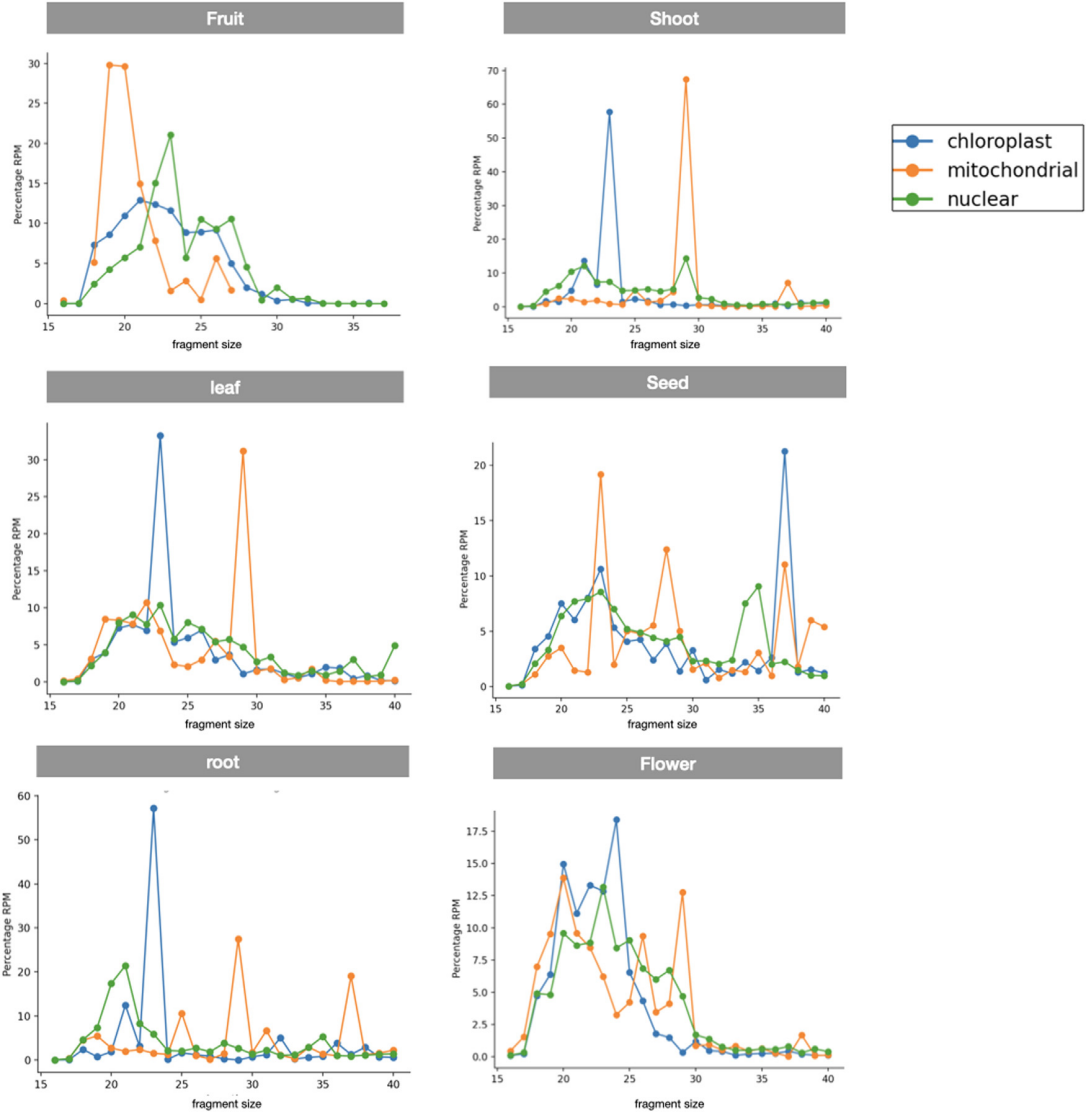
Abundance and distribution of rsRNAs in correspondence to nuclear, mitochondrial, and chloroplastic rRNAs across distinct tissue types.

**Fig. 3:**
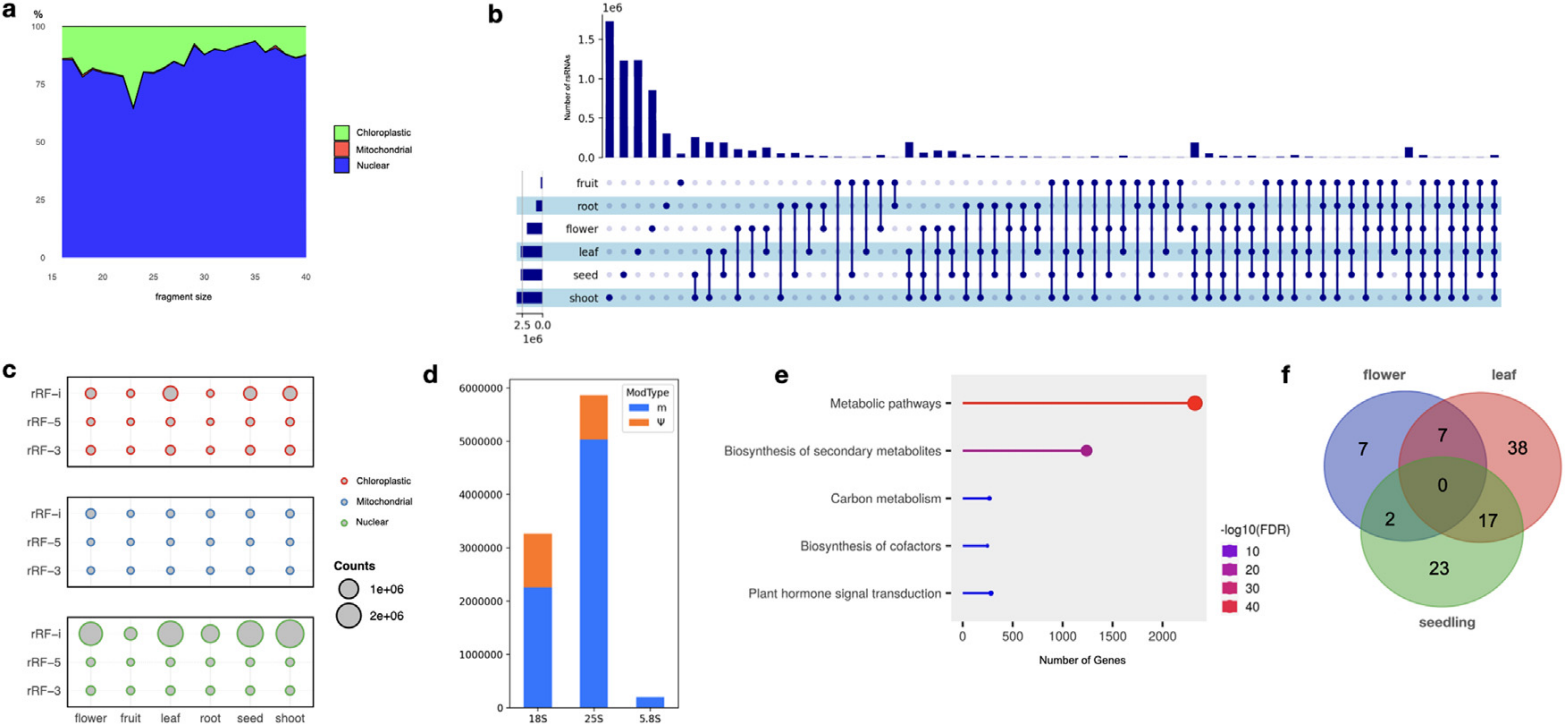
Overview of rsRNAs in *Arabidopsis thaliana.* (a) Distribution of length-wise unique annotated rsRNAs from mtrRNA, ctrRNA, and nrRNA, respectively. (b) Upset plot illustrating distinct rsRNA overlap across tissue types. (c)  Coverage of categorised rsRNA within the profiled tissues based on organellar rRNA origin. (d) Distribution of distinct methylation and pseudouridylation sites in identified rsRNAs. (e) Pathway enrichment plot of putative rsRNA targets. (f) Venn Diagram illustrating common and unique targets identified across PARE-seq datasets.

The rsRNAs annotated from our analysis show the possibility of specific fragmentation of rRNAs along with tissue-specific expression (Fig. 2, Fig. 3b). Conversely, the majority of rsRNAs displayed either widespread expression across multiple tissues or selective expression in certain tissues while being absent in others. Notably, the shoot exhibited the highest number of unique rsRNAs that were not present in any other tissue, thus, suggesting an interesting difference in enrichment of rsRNAs in distinct tissues. Further, the predominance of 5′ end rsRNAs raises questions about the role of exonucleolytic cleavage in the diversity of terminal positions, highlighting the need to investigate the dynamics of both exonucleolytic and endonucleolytic cleavage in rsRNA production similar to tsRNAs [11,[20], [21], [22]]. On top of that, the relative abundance of rsRNA types within ntRNA, mtRNA, and ctRNA space exhibited tissue-dependent variations, indicating tissue-specific differences in rsRNA abundance across different rRNA types (Fig. 3c and Figure S3). Also, the vast diversity observed in 5′ derived rsRNA implies the dynamic nature of rsRNA populations, indicating that tissue-specific factors play a significant role in their production and regulation. However, this variation as per the classification criteria is dependent on the reference rRNA in consideration. Altogether, the extensive profiling of the rsRNA expression reveals the complexities of rsRNA populations in *Arabidopsis thaliana*.

SnoRNAs play a crucial role in guiding post-transcriptional modifications of rRNA, including 2′O-methylation and pseudouridylation [23]. They are primarily localized in the nucleolus and have a diverse functional repertoire beyond rRNA modification [24], [25], [26]. A significant number of rsRNAs (Fig. 3d) contain methylation and pseudouridylation sites, suggesting the role of snoRNAs in pre-rRNA processing and to production of diverse rsRNAs. Based on the modification data and annotated snoRNAs of 25S, 5.8S, and 18S obtained from snOPY, we observed that SnoR12–1b snoRNA guides the most modifications in 25S rRNA, primarily associated with methylation rather than pseudouridylation. On the other hand, it is also important to note that there is a lack of information modification sites of rRNA in public repositories [27,16,28]. However, ongoing efforts are underway to identify modifications across all existing rRNA types. From the results, it is plausible to believe that the impact of snoRNA modifications on rRNAs extends to rsRNAs, indicating the involvement of other non-snoRNA modifications as well.

Current studies suggest that rsRNAs may also function in a manner like miRNAs (Lambert et al., 2019). Initially, we found numerous putative targets from the identified rsRNAs and further conducted gene enrichment analyses on overall identified rsRNA targets [29]. As a result, KEGG pathway enrichment analysis of rRNA-derived fragments revealed significant enrichment in multiple pathways, as shown in Fig. 3e. Among the notable pathways are metabolic pathways, biosynthesis of metabolites, carbon metabolism, biosynthesis of cofactors, and plant hormone signal transduction. These findings indicate that rsRNAs are involved in diverse metabolic processes and signaling pathways in *Arabidopsis*. Furthermore, we identified several miRNAs (ath-miR408–3p, ath-miR163, ath-miR398c-3p, ath-miR398b-3p, ath-miR398a-3p, ath-miR397a, ath-miR1888a, ath-miR396b-5p, ath-miR396a-5p, ath-miR172b-3p, ath-miR156f-5p, ath-miR400, ath-miR858a) from the miRTarBase database [30] that target some of the genes within these pathways (Table S3). These results imply additional miRNA involvement with rsRNAs, indicating a complex regulatory network that warrants further investigation. In addition, PARE-Seq analysis validated 94 rsRNA targets (Table S4) across three libraries, focusing on the top 2500 rsRNAs based on cumulative expression across sRNA-seq libraries considered in our study. Some of the targets were observed in more than one PARE-seq library. We noted that 26 targets were consistently identified in two PARE-seq libraries, underscoring the consistency of these findings across different tissue types (Fig. 3f). Collectively, this suggests that rsRNAs, beyond canonical RNAi mechanisms, likely play a role in non-canonical gene expression regulation or potentially have other unknown functions.

### Expression analyses of rsRNAs in mutant libraries

**Fig. 4:**
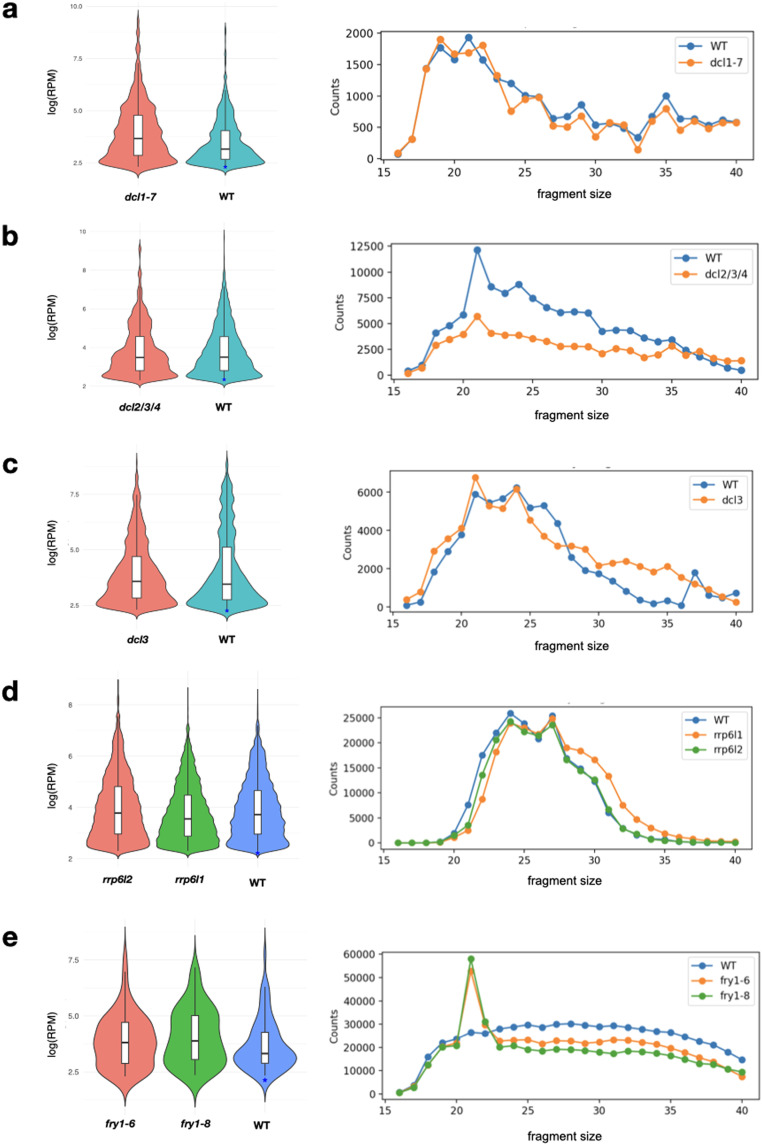
Comparison of rsRNA accumulation in mutants and WT (Col-0) of *Arabidopsis thaliana*. Distribution of rsRNAs in (a) *dcl1–7*, (b) *dcl2/3/4*, (c) *dcl3*, (d) *rrp6l1/rrp6l2*, and (e) *fry1–6/fry1–8* compared to WT, including length-wise distribution.

Upon analysis, we found that the rsRNA loci exhibited expression differences across the tested genetic backgrounds (Fig. 4). In our analysis comparing wild-type *Arabidopsis thaliana* with *dcl* and *rrp6l* mutants, we expected to observe variations in the abundance and patterns of rsRNAs. However, expression data revealed only subtle or negligible differences compared to the wild type, suggesting that the mutants had minimal impact on rsRNA abundance. Notably, we observed variations in rsRNA length distribution among the mutants, indicating potential modifications in rRNA processing and stability. For instance, the *dcl2–1 dcl3–1 dcl4–2* triple mutant, known for its more severe developmental defects compared to the wild type [31], exhibited significant alterations in the dynamics of rRNA fragmentation. To support this, a recent study highlights the significance of RDR1 and DCL2/DCL4 in the biogenesis of these rsRNAs in *hot3* mutants [32], prompting further exploration to ascertain the specific contributions of different Dicer-Like proteins in this context. These findings suggest potential differential gene expression or regulation that could influence rRNA processing, thus, indicate a direct or indirect dependency of DCL on the rsRNA biogenesis mechanism.

Furthermore, our analysis of rsRNA accumulation in the *fry1–6* and *fry1–8* mutants revealed a remarkable increase in rsRNA production, particularly in the 21 nt length, compared to the wild type. This observation points to the presence of specific mutations or genetic alterations in these mutants that affect the production of rsRNAs of particular lengths. Importantly, this observation aligns with a previous study highlighting the role of FIERY1 (FRY1) in regulating miRNA abundance by suppressing rsRNA biogenesis [33]. Thus, our results further support the involvement of FRY1 in the modulation of rsRNA levels and highlight its significance in rsRNA dynamics.

Overall, based on our current analysis, we emphasize the necessity of conducting comprehensive investigations into the roles of DCLs, FRY1, HOT3, RDRs, and other key RNAi-associated proteins in the biogenesis and molecular functions of rsRNAs in Arabidopsis and other plant species.

## Potential and existing impact

Our snakemake pipeline provides a systematic approach for detecting rsRNAs from small RNA sequencing data. This feature sets our pipeline apart from other tools due to its dedicated focus on rsRNA identification and annotation. rsRNAfinder provides a highly customizable workflow through the use of a configuration file (config.yaml). This flexibility empowers users to tailor the analysis precisely to their specific research goals and requirements. Advanced users can go even further by manipulating and extending the underlying scripts, to meet user objectives. This workflow is applicable to any species, provided that the necessary genome and rRNA files are supplied. The tool can be extensively employed in genome annotation programs to specifically annotate rsRNAs for a given species, as exemplified in the Method validation section. An additional asset of the tool is its potential applicability to studies related to understanding rsRNA biogenesis, supported by multi-omic investigations.

## Limitations and future directions

Despite its utility, rsRNAfinder faces several limitations. First, the current normalization method relies on RPM, which lacks the ability to compare rsRNA counts across different runs comprehensively. Second, the highly modified nature of rsRNAs can hinder accurate profiling during sequencing library construction, potentially impacting the precision of rsRNA landscape analysis. Third, the reference rRNA utilized is database-dependent, demanding user awareness to prevent misinterpretation; an update is planned to broaden its applicability. Fourth, the annotation of rsRNA types faces challenges due to unclear biogenesis mechanisms, limiting output accuracy. Lastly, detecting degradation of rsRNA fragments may necessitate corroborating evidence from other sources, leaving users with limited information on degradation status.

Moving forward, rsRNAfinder aims to address these limitations and push the boundaries of rsRNA research. Future developments will focus on detecting post-transcriptional modifications, potentially revealing a larger pool of significant rsRNA fragments for gene expression regulation. Improved normalization methods will be pursued to reduce noise, enhancing result accuracy, while the relationship between rsRNA expression and parental rRNA transcripts will be explored. Furthermore, rsRNAs will be classified based on their functionality, distinguishing between degraded fragments and those with regulatory roles. Finally, efforts will be directed towards faster and more accurate rsRNA gene annotation, providing functional insights alongside a better understanding of rsRNA biogenesis pathways. This holistic approach will empower researchers with a more comprehensive toolkit for exploring the world of rsRNAs in diverse species.

## Conclusion

In recent years, rsRNAs have received less attention compared to tsRNAs. Nevertheless, there is a compelling need to delve into the realm of rsRNAs and precisely identify them from small RNA-seq datasets. This would enable to understand emerging functional principles of rsRNAs along with other regulatory sRNAs. Thus, to address this research gap, our newly developed Snakemake pipeline, rsRNAfinder, provides a customizable and user-friendly approach for conducting comprehensive rsRNA analyses. This tool generates detailed reports, supports extensive annotation, and integrates a novel classification system of rsRNAs.

As an illustrative example, we leveraged extensive sRNA-seq data of *Arabidopsis thaliana* to provide insights into the tissue-specific and dynamically regulated rsRNA landscape. This analysis unveiled the unique production, high abundance, and context-specific nature of rsRNAs. Furthermore, we have also highlighted the potential involvement of the DCL and FRY in rsRNA regulation and biogenesis. It is crucial to emphasize that the rsRNAfinder tool, much like the continually evolving field of rRNA biology, remains open to ongoing refinement and maturation. However, we firmly believe that the rsRNA expression atlas generated through rsRNAfinder tool will serve as a valuable resource for fundamental research in *Arabidopsis thaliana*, enabling novel discoveries and catalyzing advancements within the realm of small RNA biology.

## CRediT authorship contribution statement

**Garima Kalakoti:** Conceptualization, Methodology, Software, Data curation, Formal analysis, Writing – review & editing. **AT Vivek:** Conceptualization, Methodology, Data curation, Visualization, Formal analysis, Writing – original draft, Writing – review & editing. **Anshul Kamboj:** Formal analysis. **Ajeet Singh:** Methodology. **Srija Chakraborty:** Formal analysis. **Shailesh Kumar:** Writing – review & editing, Validation, Supervision.

## Declaration of Competing Interest

The authors declare that they have no known competing financial interests or personal relationships that could have appeared to influence the work reported in this paper.

## Data Availability

Clone github repository (https://github.com/rebminso/rsRNAfinder.git)

## Appendix Supplementary materials

Image, application 1

Image, application 2

Image, application 3

Image, application 4

Image, application 5

Image, application 6

Image, application 7

Image, application 8
