# Supplementary material for: Comprehensive profiling of rRNA-derived small RNAs in Arabidopsis thaliana using rsRNAfinder pipeline
Source: MethodsX. 2023 Nov 25;12:102494. doi: 10.1016/j.mex.2023.102494 (PMC10711234; doi:10.1016/j.mex.2023.102494)
Supplement: Supplementary file 1 [file mmc1.docx]

**Supplementary Figure S1:** Distribution of *Arabidopsis thaliana* sRNA-seq data used in this study. (a) The read count distributions across tissue categories are depicted in sRNA-seq libraries, mapped to the *Arabidopsis thaliana genome*, and to rRNA space. (b) Comparison of mapped read percentage to the genome and rRNA space.

**Supplementary Figure S2:** Abundance plot show unique rsRNAs identified for each rRNA type.
Supplementary Figure S3 Heatmap of relative abundance of rsRNAs for each rsRNA category across tissues. For each of the rRNA types, the sum among its rsRNAs is considered to estimate relative abundance.

**Supplementary Table S1:** List of small RNA-seq datasets used to identify and annotate rsRNAs.

**Supplementary Table S2:** Mutant and corresponding wild-type small RNA libraries used in this study.

**Supplementary Table S3:** Enriched miRNA pathways co-associated with identified rsRNAs.

**Supplementary Table S4:** List of validated rsRNA targets**.**
