# Supplementary figures and images for: Comprehensive profiling of rRNA-derived small RNAs in Arabidopsis thaliana using rsRNAfinder pipeline
Source: MethodsX. 2023 Nov 25;12:102494. doi: 10.1016/j.mex.2023.102494 (PMC10711234; doi:10.1016/j.mex.2023.102494)

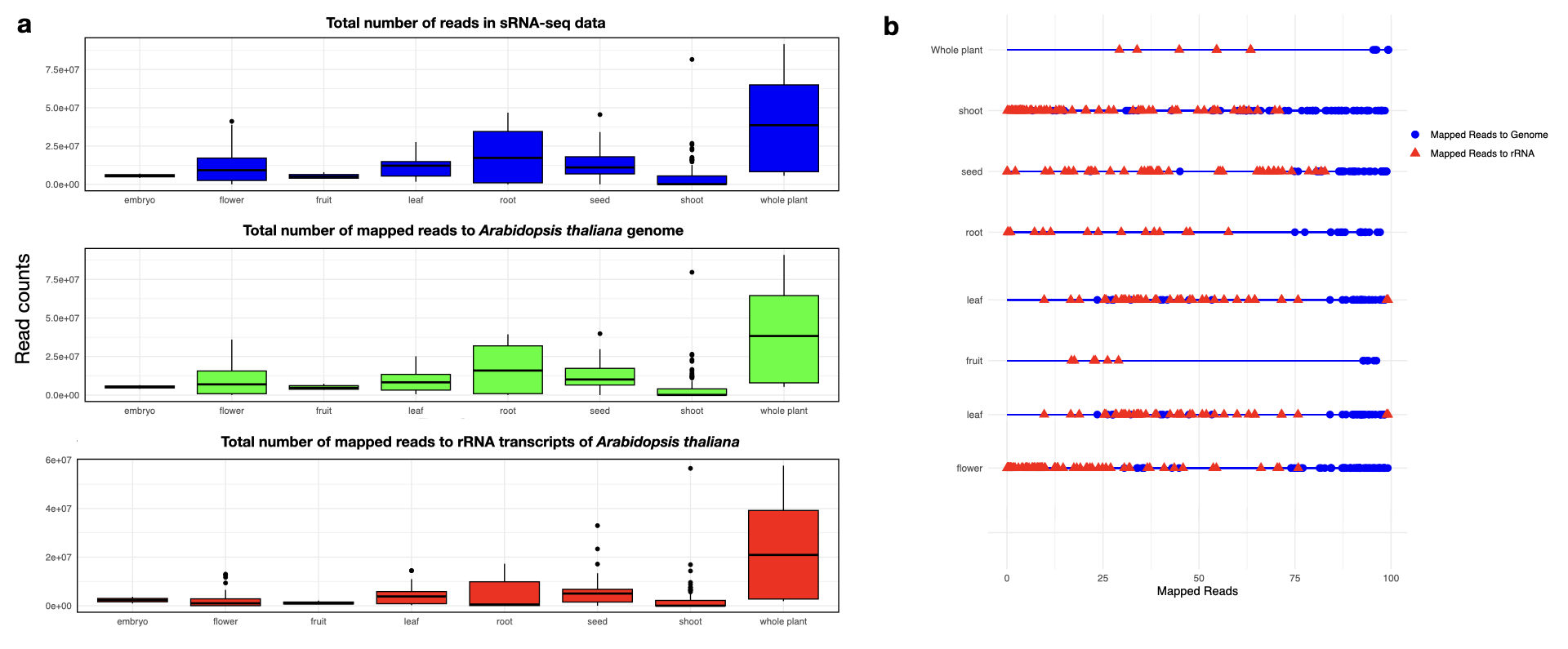

Supplement: Supplementary file 2 [file mmc2.zip › mmc2.tiff]

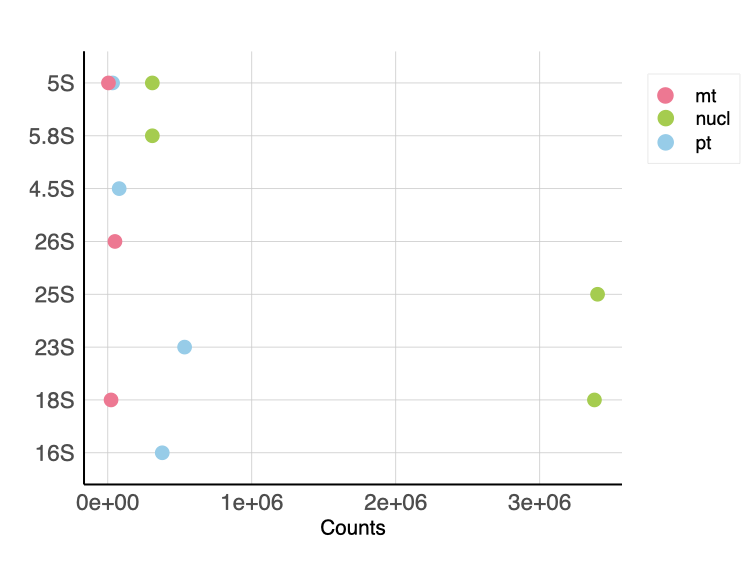

Supplement: Supplementary file 3 [file mmc3.zip › mmc3.tiff]

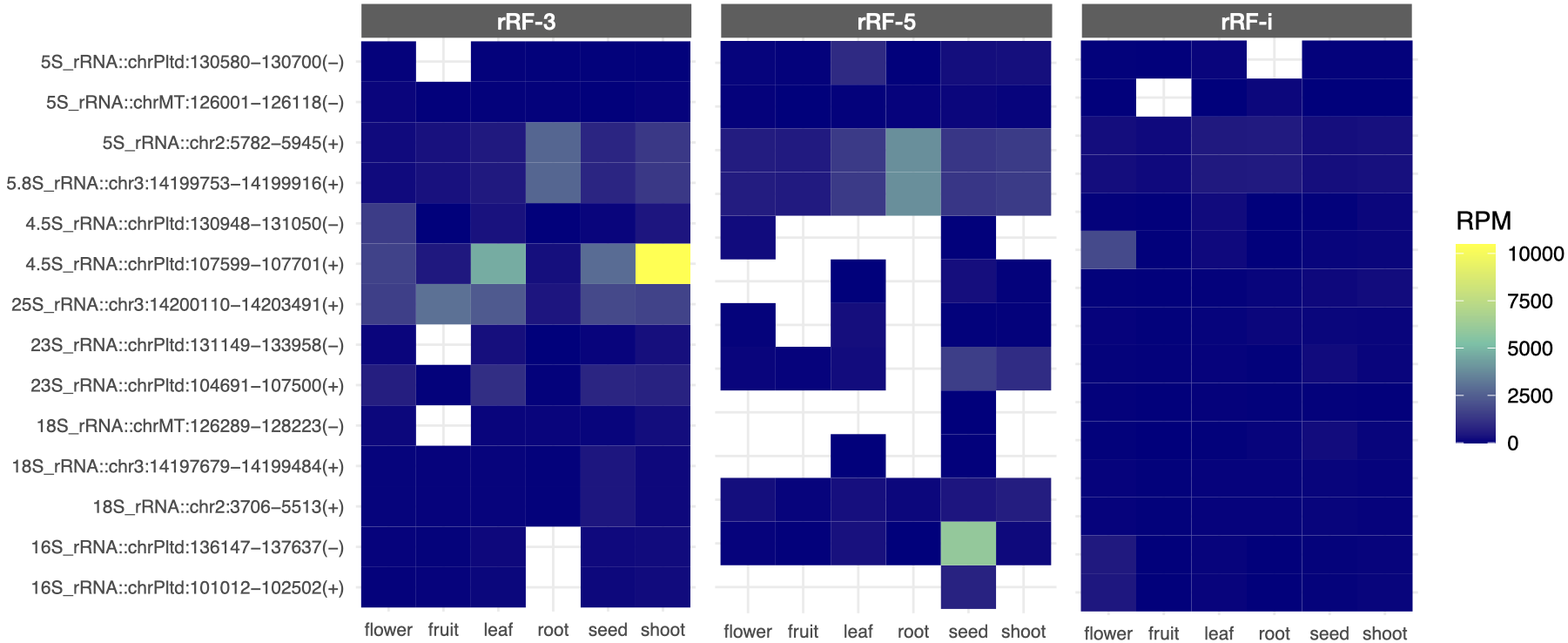

Supplement: Supplementary file 4 [file mmc4.zip › mmc4.tiff]
